# Andean and California condors possess dissimilar genetic composition but exhibit similar demographic histories

**DOI:** 10.1002/ece3.6887

**Published:** 2020-10-21

**Authors:** Julian Padró, Sergio A. Lambertucci, Paula L. Perrig, Jonathan N. Pauli

**Affiliations:** ^1^ Grupo de Investigaciones en Biología de la Conservación INIBIOMA, Universidad Nacional del Comahue ‐ CONICET Bariloche Argentina; ^2^ Department of Forest and Wildlife Ecology University of Wisconsin‐Madison Madison WI USA

**Keywords:** ancient DNA, bottleneck, genetic diversity, museum, scavenger, vulture

## Abstract

While genetic diversity of threatened species is a major concern of conservation biologists, historic patterns of genetic variation are often unknown. A powerful approach to assess patterns and processes of genetic erosion is via ancient DNA techniques. Herein, we analyzed mtDNA from historical samples (1800s to present) of Andean Condors (*Vultur gryphus*) to investigate whether contemporary low genetic variability is the result of recent human expansion and persecution, and compared this genetic history to that of California condors (*Gymnogyps californianus*).We then explored historic demographies for both species via coalescent simulations. We found that Andean condors have lost at least 17% of their genetic variation in the early 20th century. Unlike California condors, however, low mtDNA diversity in the Andean condor was mostly ancient, before European arrival. However, we found that both condor species shared similar demographies in that population bottlenecks were recent and co‐occurred with the introduction of livestock to the Americas and the global collapse of marine mammals. Given the combined information on genetic and demographic processes, we suggest that the protection of key habitats should be targeted for conserving extant genetic diversity and facilitate the natural recolonization of lost territories, while nuclear genomic data should be used to inform translocation plans.

## Graphical abstract

Andean condors have lost at least 17% genetic variation by the early 20th century. Demographic bottlenecks are associated with global anthropogenic processes, however, unlike California condors, low mtDNA diversity in Andean condors is mostly ancient. Conservation measures require to include both environmental and genetic management. Photo credit: Gonzalo Ignazi.

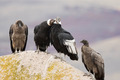

## 1 INTRODUCTION

Genetic diversity is widely recognized as centrally important for biodiversity assessment and conservation planning (Willoughby et al., 2015). Such focus is especially important when considering threatened and endangered species as they typically display low levels of genetic diversity, which can lead to reduced adaptive potential to environmental change (Bijlsma & Loeschcke, 2012). A common strategy to restore genetic diversity is the translocation of individuals to augment small and declining populations (Moritz, 1999; Whiteley et al., 2015). However, the motivation for restoring genetic diversity is often based on genetic surveys of modern populations, which assumes that past genetic diversity was always higher (Matocq & Villablanca, 2001; Napier et al., 2020). Nevertheless, long‐term effects of translocations have been rarely assessed, and findings are inconsistent across systems: Some translocations have succeeded in increasing population fitness by incorporating new alleles (Johnson et al., 2010; Whiteley et al., 2015), while others have failed by underestimating the negative effects of genetic homogenization, outbreeding depression, disease transmissions or behavioral divergence (e.g., Deredec & Courchamp, 2007; Grauer et al., 2017; Kock et al., 2010; Manlick et al., 2017). This points to the importance of considering the natural history of populations in management plans, especially when species are geographically widespread as they may exhibit considerable differences in the degree of adaptation, isolation, and gene flow, resulting in possible incompatible genetic traits (DiBattista et al., 2020; Moritz, 1999).

One powerful approach is to harness DNA from museum collections to study the historical genetic composition of populations and understand both the spatial and temporal changes experienced (Wandeler et al., 2007). For example, recent studies have utilized museum specimens to reconstruct historic colonization routes (Pauli et al., 2015) and genetic structure (Black et al., 2018), quantify the degree of genetic erosion in persisting species (Hailer et al., 2006), and evaluate the effectiveness of reintroductions (Godoy et al., 2004).

A useful comparison within conservation biology is the management of the two extant species of condors, the Andean condor (*Vultur gryphus*) and the California condor (*Gymnogyps californianus*). The latter has been an object of intense genetic studies due to its near extinction in the 1980s and its subsequent breeding program in the 1990s (Snyder & Snyder, 2000). The first studies aimed at assisting in management decisions of the captive population revealed low levels of genetic variability (Ralls & Ballou, 2004). Additional analysis involving historic samples from the past two centuries confirmed that current diversity levels are a direct consequence of recent human persecution, resulting in >80% genetic decline (D'Elia et al., 2016), and currently, whole genome sequencing is being used for guiding smart breeding programs (Ryder et al., 2016). In contrast, the genetics of Andean condors has received little attention despite population declines. Though Andean condors remain widespread across South America, ranging throughout the Andean mountains from Colombia to the southernmost headlands of Argentina and Chile, they have undergone notable declines in distribution and abundance (BirdLife International, 2017; Plaza & Lambertucci, 2020). Indeed, in the early 20th century, Andean condors once were distributed as far north as Venezuela (Calchi & Viloria, 1991), while historical records from expeditions in the early 19th century indicate that the Andean condor once inhabited the Atlantic coasts of Patagonia (Darwin, 1841; Hatcher, 1903). However, human prosecution during European colonization precipitated a continental‐scale decline, resulting in local extinctions and retraction across its distributional range. In 1970, the species was declared “Endangered” (USFWS, 1970), and three decades later was listed as globally “Near Threatened” by the International Union for Conservation of Nature (BirdLife International, 2017) and is currently recommended to be listed as “Vulnerable” (Plaza & Lambertucci, 2020). At the local level, the species is “Critically Endangered” in its northern distribution: Condors are functionally extirpated in Venezuela, <150 individuals inhabit Colombia, ~100 individuals inhabit at Ecuador, ~250 to 1,000 remain in Bolivia, and ~300 to 2,500 are estimated to occupy Peru (Méndez et al., 2019; Naveda‐Rodríguez et al., 2016; Piana & Angulo, 2015). In the southern part of its distributional range, Andean condors were extirpated from the steppe and the Atlantic coasts 100 years ago (Conway, 2005), but holdout in the high Andes (Lambertucci et al., 2018; Perrig et al., 2017, 2020). These extant populations continue to decline due to persistent poaching and habitat degradation, and face new challenges such as dietary toxins (especially lead and pesticides) and collisions with power lines (Alarcón & Lambertucci, 2018; Pavez & Estades, 2016; Wiemeyer et al., 2017).

Conservation of condors has primarily focused on programs aimed to reinforce extant populations and repopulate extinct ones via human‐assisted translocations (Astore et al., 2017; Snyder & Snyder, 2000). The first releases of Andean condors occurred in Colombia in 1989 using captively reared birds (Lieberman et al., 1993). This was followed by subsequent translocations in Colombia, Venezuela, Chile, Bolivia, and Argentina and, notably, recent efforts to repopulate the historic range along the Atlantic coast (Astore et al., 2017). Many of the released condors were translocated from distant regions or from undocumented sources. For instance, several translocations used condor offspring from central Argentina to reinforce Chile and repopulate Venezuela and the Atlantic coast. As a result, >229 Andean condors have been released in South America to date (Astore et al., 2017) and future translocations are being considered in new regions, including Ecuador (Naveda‐Rodríguez et al., 2016). However, management plans considering the historic genetic composition of natural populations are lacking and are limited to a small mitochondrial dataset that revealed low levels of genetic variation (Hendrickson et al., 2003). More recently, however, we have shown that condors exhibit nuclear genetic diversity levels similar to those of other widely distributed vultures and present some degree of genetic differentiation across regions at the core distribution of the species (Padró et al., 2018, 2019). Thus, it is unclear if the low genetic variability found at the continental scale is the result of low sample size, recent demographic declines, and range contraction or due to an ancestral state of mtDNA.

Herein, we investigated the evolution of genetic diversity of Andean condors across their entire historic range. We employed historical mtDNA from museum specimens and genetic data from contemporary populations to evaluate whether the observed low genetic diversity is ancient and potentially intrinsic to the species or, as in the case of the California condor, is related to the recent population decline and contraction of its distributional range. We also employed coalescent simulations to evaluate the existence and intensity of major demographic bottlenecks in both condor species to assess whether similar processes have shaped the demographic dynamics of these vultures exhibiting similar ecological and life‐history traits and that face similar threats.

## 2 Methods

### 2.1 Sample collection

We obtained DNA samples of 42 Andean condors from eight museums in the United States and Argentina, collected between 1884 and 1970, spanning the historic distributional range of the species, including the now extirpated populations of Venezuela and eastern Patagonia. We also included five modern samples collected between 2008 and 2013 from the south, north, and central mountains of Argentina to represent contemporary haplotypes. Finally, we obtained previously published mtDNA dataset from 32 individuals collected across the continent between 1961 and 1999 plus five individuals collected between 1932 and 1946 (Hendrickson et al., 2003). The temporal distribution of the combined dataset exhibited clear bimodality, with a breakpoint between historical (1884–1946) and contemporary samples (1961–2013). The collection dates of historical samples occurred either before or during the decline and range contraction of Andean condors. Specifically, by the first half of the 20th century the species was considered extinct in Venezuela (Calchi & Viloria, 1991) and was disappearing in Colombia (Lieberman et al., 1993; Márquez et al., 2005), and the last sightings of condors nesting on the Atlantic coast were made (Adams, 1907). Given that condors can live up to 70 years of age (Kasielke & Wallace, 1990; Meretsky et al., 2000), our historical time frame does not include individuals from the current generation.

### 2.2 Molecular techniques

We extracted historic DNA from several types of tissues including feathers, toe pads, skin snips, and bone fragments using a modified QIAamp kit protocol (Qiagen) by incorporating 10 μl 1 M of dithiothreitol (DTT) and 3 μg of RNA carrier to the digestion buffer. Contemporary DNA was extracted from molted feathers collected from roosting sites in central Argentina (see Padró et al., 2018). All extraction procedures were conducted in an isolated pre‐PCR ultraclean room, equipped with air filtering units and UV lights dedicated to low‐template DNA analysis. As a precaution, we handled no more than 10 samples per batch, and in addition to negative controls, we also performed a subset of replicate DNA extractions in each batch to detect possible contamination.

To perform the combined analyses with the previous dataset, we targeted mtDNA at the Control region and 12S rRNA. We initially amplified ~600 bp of the Control region and ~360 bp of 12S using the primers designed for Andean condors L16652‐H621, L798‐H1455, and L798‐H1795 (Hendrickson et al., 2003). Degraded DNA in our historical samples (average fragments of ~200 bp) required us to design primers spanning an internal segment of 145 and 165 bp containing the segregating sites at positions 817 and 851 of Control region and positions 1,199 and 1,318 of 12S, hence covering most of the reported genetic variation of Andean condors (VGaDNACRF2 5′‐CAAGAATCCCTGAATGAGACG‐3′; VGaDNACRR2 5′‐GGTAGTAAATCATGTCCAACAAGC‐3′; VGaDNA12SF1 5′‐GCAATAAGTGTAAACTTGACTTAGT‐3′; VGaDNA12SR1 5′‐TTATGACAGCTCAGTTACGG‐3′). PCR amplifications were carried out in 10 or 25 μl reactions using 1x PCR Buffer with 0.02 U/µl of Q5 high‐fidelity DNA polymerase (New England Biolabs), 0.6 µM of each forward and reverse primer, 200 µM of dNTPs and 30% BSA. PCRs began with 5‐min denaturation at 94°C, followed by 38 cycles of 30‐s denaturation at 94°C, 30‐s annealing at 60°C, and 30‐s extension at 72°C, followed by 10 m of final extension at 72°C. PCR products were purified with ExoSAP‐IT (USB) and sequenced in an ABI 3730xl DNA Analyzer (Applied Biosystems) in both 5′ and 3′ directions two times to test for possible genotyping errors. Resulting sequence chromatograms were visualized and aligned in MEGA‐X using the MUSCLE algorithm (Kumar et al., 2018).

### 2.3 Genetic diversity analysis

**Figure 1:**
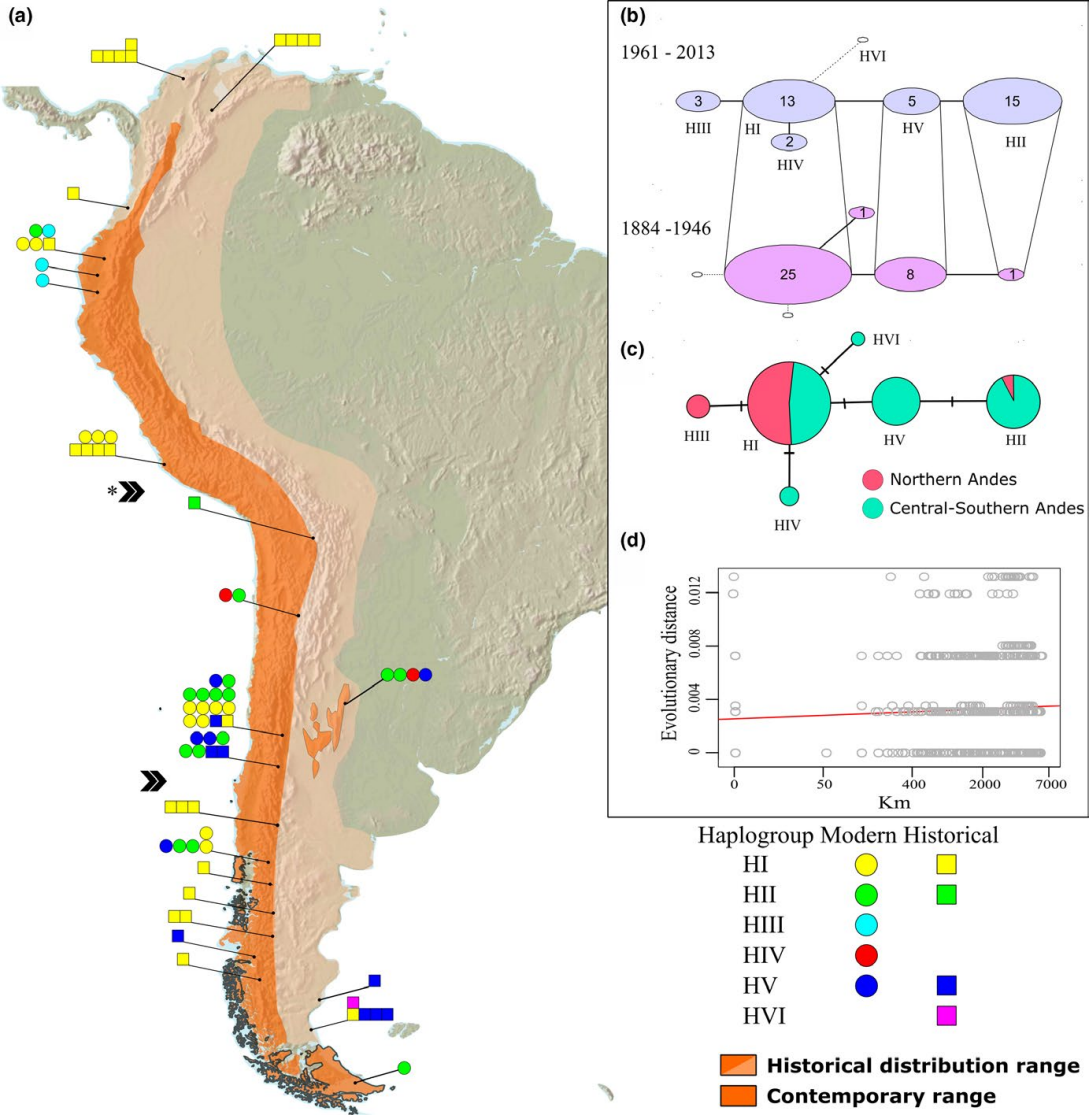
Continental distribution of historical (1884–1946) and contemporary (1961–2013) mtDNA haplotypes of *Vultur gryphus* (a). Divisions of putative populations from northern, central, and southern Andes are marked (»; * denote significant geographic differentiation). Temporal network (b) is represented in two chronological time layers linked by haplotype continuity (empty circles symbolize missing haplotypes for that time period while numerals denote sample size). Spatial network (c) depicts the proportion of haplotypes present in each region (roman numbers denote haplogroups). Scatter plot (d) of isolation by distance of 73 individuals sampled across South America

We combined our historical and contemporary data with previously published sequences (GenBank accession numbers in Table S1) and trimmed and aligned all sequences with our short reads. We then collapsed Control region (145 bp) and 12S (165 bp) sequences into a single 310 bp haplotype using FaBox (Villesen, 2007) to construct a temporal haplotype parsimony network and calculate the statistical probability of each link being evolutionarily correct using the *TempNet* script (Prost & Anderson, 2011) in R (R Core Team, 2017). We inspected the consistency of the haplotype network by considering mutation rate heterogeneity between coding and noncoding regions in NETWORK 4.6 (Bandelt et al., 1999), giving smaller weights to nucleotide positions in the Control region. We then calculated haplotype diversity (*H*), nucleotide diversity (*π*), and the average number of nucleotide differences (*k*) in DnaSP 6.0 (Rozas et al., 2017). We assessed the extent of genetic differentiation between regions and time periods with φST pairwise comparisons (10,000 permutations) and with exact tests of population differentiation using 1,000,000 Markov chain steps and 500,000 dememorization steps as implemented in ARLEQUIN 5.3.2 (Excoffier & Lischer, 2010). Our analyses were performed on three regional groupings of samples based on dispersal expectations of condors. We defined a priori populations by geographic proximity according to the species’ gene‐flow distance that has been recently estimated as a radius of ~1,200 km of each putative population (Padró et al., 2018), dividing northern, central, and southern Andes, providing us with a balanced sample size of 24, 28, and 21 individuals, respectively (Figure 1a). We then employed hierarchical analyses of molecular variance to evaluate the amount of population genetic structure across time periods for the whole continent and among regions within time periods using 10,000 nonparametric permutations. Finally, we employed Mantel tests to assess the correlation between the evolutionary genetic distance, taking into account differences between nucleotides and inequality of frequencies (maximum composite likelihood method; Kumar et al., 2018), and geographic distances (10,000 permutations) using *adegenet* R package (Jombart & Ahmed, 2011).

### 2.4 Demographic analysis

**Figure 2:**
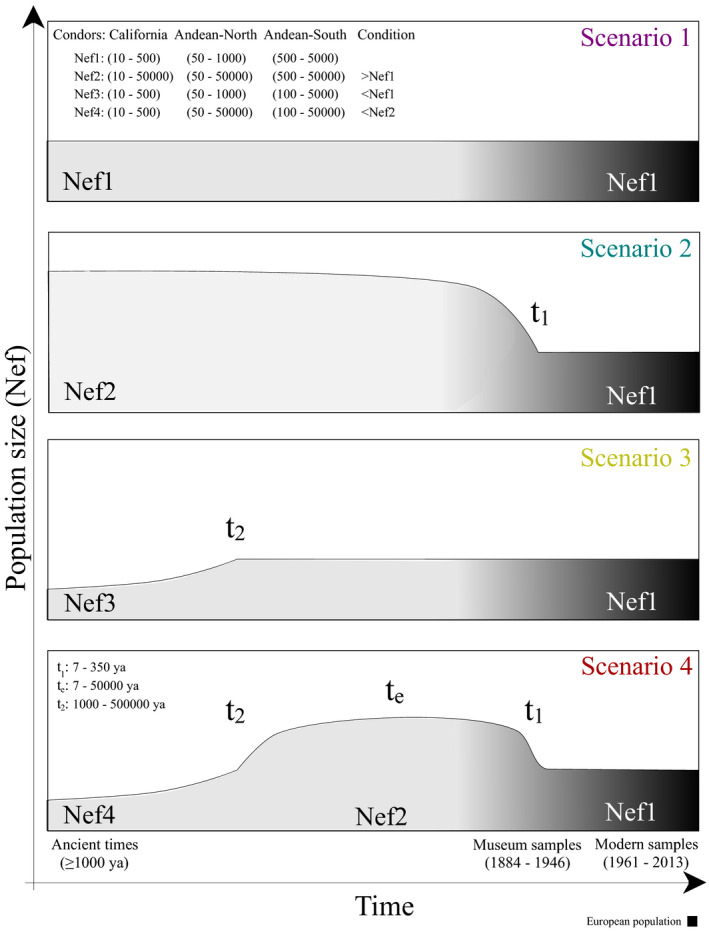
Diagram of four demographic scenarios modeled for California and Andean condors, using Approximate Bayesian Computation. General model parameters: Nef_x_ denotes the female effective population size and *t*_x_ stands for the timing of demographic events

To explore the regional demographic history of condors, we performed approximate Bayesian computation analysis in DYABC 2.1.0 (Cornuet et al., 2014). This coalescent‐based approach allowed us to test alternative bottleneck scenarios that may have acted on natural populations by comparing the summary statistics of the simulated and empirical data. We performed simulations including the historical and contemporary samples from the North (15 historic and 9 contemporary individuals from Venezuela, Colombia, Ecuador, and Peru) and Central‐South (20 historic and 29 contemporary individuals from Bolivia, Chile, and Argentina) of South America (see Figure 1a). We also performed simulation analysis in the California condor using 65 historical and 14 contemporary mtDNA sequences (569 bp of Control region) from western Mexico and United States (D'Elia et al., 2016; Table S2). Both species typically begin breeding at 6–8 years of age (Houston, 1994; Snyder & Snyder, 2000); we set the generational time at 7 years. The mutational model of each locus was determined by the likelihood scores in JModelTest 2.1.10 (Darriba et al., 2012), resulting in HKY for both Andean and California condors (parameter details in Table S3). We modeled four different scenarios for the observed patterns of genetic variability in condors (Figure 2). First, we introduced the no‐change scenario, assuming no significant variation in population size across time (Figure 2; Scenario 1). Given that both species of condors experienced populations declines and range contractions during the last centuries, we also simulated a large ancient population size with an historical‐recent bottleneck scenario, using a prior timing uniformly distributed between 7 and 350 years ago (*t*_1_) to take into account the possible influence of the early European settlers and modern civilization (Figure 2; Scenario 2). Populations could also have declined as the consequence of historic climatic changes or the extinction of megafauna during the late Pleistocene (Emslie, 1987; Tambussi & Noriega, 1999). Thus, we included an ancient bottleneck uniformly distributed between 1,000 and 500,000 years ago (*t*_2_) without the historical‐recent bottleneck (Figure 2; Scenario 3). Given that Andean and California condors are the only surviving members of a once diverse guild, it is possible to consider that the extinction of competitors during the late Pleistocene (>10,000 years ago) favored some demographic boost (Emslie, 1988; Perrig et al., 2019; Tonni & Noriega, 1998) or condors initially benefited from the introduction of livestock in the Americas before population declines (Adams, 1907; Emslie, 1987). Thus, we included a scenario with an expansion time period uniformly distributed up to 50,000 years (*t*_e_) between the ancient and historical‐recent bottlenecks (Figure 2; Scenario 4).

We set the prior information of the historic‐recent effective female population size (Nef1) of Andean condors between 50 and 1,000 for the northern region and between 500 and 5,000 for the south. These priors were based on the estimates of regional population censuses of <2,570 individuals from Peru to Venezuela, >3,000 between Bolivia, Chile, and Argentina, and an estimated total number of 6,700 mature individuals in the whole continent (BirdLife International, 2017; Naveda‐Rodríguez et al., 2016). Given that in 1987 California condors were comprised by 27 individuals (Ralls & Ballou, 2004) and had an estimated population of ~150 individuals by the middle of the 20th century (D'Elia et al., 2016), we established a lower bound of 10 and an upper limit of 500 individuals, allowing for a larger population size before the mid‐20th century. Population size during the expansion period (Nef2) was conditioned to be larger than Nef1, while population size in the ancient bottlenecks Nef 3 and Nef4 was estimated to be lower than Nef1 and Nef2, respectively (Figure 2; details in Table S4). After running 5 × 10^6^ simulations for each analysis, we pre‐evaluated the prior distributions by comparing the distribution of summary statistics and observed data using principal component analysis. We choose the most likely scenario based on their posterior probability applying polychotomous logistic regressions of each scenario probability on the deviations between simulated and observed summary statistics (based on 1% of simulated datasets) and comparing their 95% confidence intervals (Cornuet et al., 2014). We then calculated the posterior distribution of parameters in the most likely scenario by local linear regressions using 1% of simulated data closest to the observed data. Finally, we evaluated confidence in the scenario by calculating type I and II errors and performed model checking computations to assess the “goodness‐of‐fit” by comparing the observed data with the 1% of simulated data under each model‐posterior combination (Cornuet et al., 2010).

## 3 RESULTS

### 3.1 Genetic diversity

We sequenced Control and 12S regions from 33 historical and 8 contemporary samples (41 samples), including five samples previously amplified by Hendrickson et al. (2003) that matched our sequences. Our historical samples were comprised by three haplogroups (HI, HII, and HV) occurring in modern samples and previously reported, plus a unique haplotype (HVI) found in an historical sample of 1896 from the extirpated population along the Patagonian coast, exhibiting novel T‐C transitions at base‐pair positions 817 and 851 of the Control region (archived in GenBank under the accession number MT993980). We also obtained six partial haplotypes (Control or 12S regions) from four historic and two contemporary samples, all showing no diagnostic or novel substitutions that were excluded from further analysis. The combination of our samples with those amplified by Hendrickson resulted in a final number of 73 samples from 35 historical and 38 contemporary individuals, comprising six haplogroups distributed across the entire distribution range of Andean condors (Figure 1a; Table S1).

Our temporal haplotype network revealed the presence of four haplotypes (HI, HII, HV, and HVI) in the historic samples and five haplotypes (HI‐V) in contemporary populations, resulting in the loss of one haplotype (HVI) in the 20th century, or a 17% of decline (Figure 1b). All links in the haplotype network exhibited > 99.8% of probabilities in their evolutionary links, indicating that the haplogroups did not diverge through a third haplotype. Pairwise comparisons of φST and exact tests of differentiation between regions showed that most of the genetic structuring was due to differences of the north against the central (φST: 0.55, *p* < .001, exact‐*p* = .01) and southern Andes (φST: 0.16, *p* < .01, exact‐*p* < .01), but not between central and south (*p* > .05 in all cases). The spatial sorting of the haplogroups between central‐southern (Bolivia, Chile, and Argentina) and northern Andes (Peru, Ecuador, Colombia, and Venezuela) showed that HIII was exclusively found in the north; HIV, HV, and HVI were only present in the south, while HII was predominant in the south (93.75%) and HI was evenly distributed across regions (Figure 1c). Mantel test between genetic and logarithmic geographic distance revealed significant isolation by distance (*n* = 73, *r* = .07, *p* = .02), indicating a history of evolutionary divergence between north and south (Figure 1d). Nucleotide and haplotype diversity levels were higher in the contemporary samples and in central‐southern South America (Table 1). Our comparisons of φST and exact tests between time periods showed significant temporal structure (φST: 0.17, *p* < .01, exact‐*p* = <.001). However, our hierarchical AMOVA showed no statistical differences across time, while regions exhibited marked differences within time periods, with most of the genetic variation explained within regions (Table 2).

**Table 1:** Genetic diversity of mtDNA of Andean condors across time and between regions of South America

Time period/ Region	*n*	*n_H_*	π (*SD*)	*H* (*SD*)	*K* (var)
Historical	35	4	0.0016 (0.0004)	0.449 (0.083)	0.504 (0.193)
Contemporary	38	5	0.0040 (0.0003)	0.720 (0.043)	1.255 (0.656)
Northern Andes	24	3	0.0013 (0.0006)	0.301 (0.112)	0.395 (0.146)
Central‐Southern Andes	49	5	0.0033 (0.0002)	0.713 (0.026)	1.029 (0.489)
Overall	73	6	0.0032 (0.0003)	0.656 (0.043)	0.982 (0.453)

Note

Sample size (*n*); number of haplotypes (*n_H_*); nucleotide diversity (*π*); haplotype diversity (*H*); average number of nucleotide differences (*K*).

**Table 2:** Results of hierarchical AMOVA for Andean condor individuals based on mtDNA haplotypes

Source of Variation	*df*	Sum of squares	Var Comp	% of variation	*F*‐statistic	*p*‐value
Among time periods	1	2.658	0.019	5.36	FCT = 0.054	>.668
Among regions within time period	2	3.022	0.081	22.5	FSC = 0.237	<.001
Within regions	69	17.923	0.256	72.14	FST = 0.279	<.0001
Total	72	23.603	0.36			

### 3.2 Demographic scenarios

**Figure 3:**
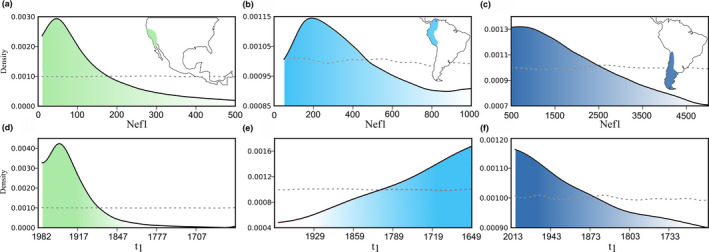
Prior (dash line) and posterior distribution (solid line) of historic effective population size and timing of bottleneck (*Anno Domini;* assuming a 7‐year generation time) for California condors (a, d) and Andean condors from north (b, e) and central‐south of South America (c, f)

Our pre‐evaluation analysis (prior definitions) and comparison of posterior probabilities between scenarios showed consistent results, supporting scenario 4 in California and Andean condors from central‐south of South America, whereas scenario 2 was the most supported in northern Andean condors. Principal component analysis revealed that the observed data fitted well within the simulated prior combinations of the supported scenarios and comparatively similar or better than in the alternative models (Figure S1). Posterior probabilities of the supported scenario were higher in California condors (*Pr* = 0.568, CI: 0.562–0.573) and southern Andean condors (*Pr* = 0.540, CI: 0.532–0.549) with 95% confidence intervals not overlapping with alternative scenarios. Northern Andean condors showed lower posterior probabilities (*Pr* = 0.436, CI: 0.432–0.440), although with no confidence interval overlapping with other scenarios (details in Table S5). Measures of central tendency of the posterior distribution of the best model in the California condor showed an historic effective population size between 37 and 142 (Nef1), with a recent bottleneck during 1918–1940 (*t*_1_), and an ancient population size of 32,200–47,400 (Nef2) between 2,576 and 23,730 years ago (*t*_e_) and a lower population size reaching 853–14,300 (Nef4), 52,500–227,500 years ago (*t*_2_). Historical population size in northern Andean condors was 170–503 (Nef1), with an historical bottleneck around 1662–1781 (*t*_1_), and an ancient population size of 6,760–15,900 (Nef2). Historical population size of southern Andean condors was 855–2,320 (Nef1) after a bottleneck around 1844–1963 (*t*_1_), harboring an ancient population size between 39,700–47,100 individuals, 1,792–20,020 year ago, prior a lower population size between 22,300–33,200 individuals, 329,000–452,900 years ago (distributions of historic parameters in Figure 3; details in Table S6). Our analyses showed that type I error rate was 0.35, 0.61, and 0.39 for California and Andean condors from the north and central‐south, respectively. Notwithstanding, most of the error rates were explained by miss‐classification between scenarios 2 and 4, both including recent demographic bottlenecks (Figure 2; Table S7), and a combined posterior probability > 99% for California and southern Andean condors and > 71% in northern Andean condors (Table S5). Type II error was 0.16 for the California condor, whereas in the Andean condor was 0.12 and 0.23 for the north and central‐south, respectively. Our assessment of the goodness‐of‐fit (model checking) showed that the observed datasets fitted well into the posterior predictive distributions of the supported scenarios, indicating that the chosen model parameters correctly explain the data summarized by summary statistics in both California and Andean condors (Figure S2).

## 4 DISCUSSION

### 4.1 Genetic diversity patterns

Our study of contemporary and historical mitochondrial data in conjunction with a continental‐level sampling of the Andean condor allowed us to characterize the spatiotemporal genetic composition for this species of special conservation concern. We showed that Andean condors have lost at least 17% of genetic variation by the early 20th century. As expected, genetic erosion was associated with the loss of a mitochondrial lineage in the peripheral distribution during the contraction of its southern geographic margin in the Atlantic coast. However, we found no evidence of lost haplotypes in the northern distribution where populations have been driven to near‐extinction (Plaza & Lambertucci, 2020; Naveda‐Rodríguez et al., 2016). A possible explanation is that the genetic decline and range contraction in the north may have started long before the collection dates. Lower genetic variation and detection of significant mitochondrial substructure against the central‐southern region reinforce the idea of genetic drift acting in northern South America.

Overall, our study yielded similar results to those reported by Hendrickson et al. (2003), indicating that the extreme low mtDNA diversity in the Andean Condor is mostly ancient. In fact, we found more genetic variation in the contemporary samples than historical ones, resulting in mixed results between time periods. Low levels of genetic diversity seem to be intrinsic to the life‐history traits of many raptors, as long generational times result in low mutation rates, large body size often reflects smaller net effective population sizes, and high dispersal power may result in homogenizing effects (Berlin et al., 2007; Martínez‐Cruz, 2011). For example, the Cinereous vulture (*Aegypius monachus*), the red kite (*Milvus milvus*), and the Spanish Imperial eagle (*Aquila adalberti*) exhibit one of the lowest mtDNA diversity levels with ≤ 10 haplotypes reported for the Control region (Martínez‐Cruz et al., 2007; Poulakakis et al., 2008; Roques & Negro, 2005), while California condors possess only three haplotypes (D'Elia et al., 2016). However, while such genetic impoverishment has been interpreted to be exacerbated by founder events or bottlenecks during periods of climate change in the first three species, current diversity pattern in the California condor is explained by direct human prosecution, resulting in more than 80% genetic decline.

We found that although current levels of genetic diversity in the Andean condor are related to the extirpation of peripheral populations, diversity was already low when compared to the historic levels of California condors. We cannot, however, rule out a potential effect of the difference in the sequences of the historical samples between California condors (569 bp from control region) and Andean condors (310 bp from 12S and Control region). However, if this pattern is indeed species‐specific, factors other than life‐history traits should be invoked to explain such disparity in the mutation rates between condor species. For instance, it is possible that species have experienced disparate evolutionary histories during phylogenetic radiation such as contrasting levels of ancestral genetic variation, sex‐biased asymmetries, different strengths of genomic sweeps, introgression events, or mito‐nuclear conflicts (e.g., Berlin et al., 2007; Dowling et al., 2008; Toews & Brelsford, 2012). Many authors have stressed the importance of natural selection in shaping divergent patterns of mtDNA across species, including the role of selection in mito‐nuclear fitness interactions, where depending on the variation in the nuclear genome could constrain mitochondrial variation (e.g., Dowling et al., 2008). In addition, demographic and behavioral factors can also promote mtDNA homogenization in systems with skewed sex ratios or female‐biased dispersal (Toews & Brelsford, 2012). Although Andean condors do not exhibit sex‐biased dispersal (Padró et al., 2018), skewed sex ratios have been suggested to be driven by higher female mortality (Lambertucci et al., 2012; Plaza & Lambertucci, 2020), possibly promoting mtDNA homogenization. Moreover, as observed among Cinereous vultures (Poulakakis et al., 2008) and Spanish Imperial eagles (Martínez‐Cruz et al., 2007), Andean condors from central Argentina exhibited low mtDNA diversity, but show no evidence of nuclear genome‐wide erosion (Padró et al., 2018), suggesting that evolutionary constraints on genetic diversity only affected the mitochondrial genome.

### 4.2 Demographic processes

In concordance with previous work, we found that California condors and southern Andean condors experienced ancient demographic expansions, probably due to the abrupt reduction in competition with sibling species during the megafauna extinction in the Pleistocene era (Perrig et al., 2019). The lack of detection of ancient bottleneck in northern Andean condors might be explained by low statistical power due to the reduced genetic variability or because of older coalescence times compatible with the northern origin of the group (Emslie, 1988; Tambussi & Noriega, 1999). If true, demographic expansion in the central‐south could also be attributed to the recolonization dynamic from the glacial refugia in the tropic (Hewitt, 2000). Our estimated timing of demographic expansion (1,792–20,020 ya) is consistent with the postglacial recolonization of higher latitudes by highly vagile species of the southern hemisphere such as whales, seals, and penguins (Fraser et al., 2012; Thatje et al., 2008). Regardless of the specific demographic processes involved in the ancient past, it is clear that all extant condor species experienced recent mtDNA bottlenecks.

Our estimated dates for the recent‐historic bottlenecks in Andean condors are in concordance with the timing of European colonization in South America. The early historic bottleneck in northern Andean condors matches the economic growth of livestock production during the 17th and 18th centuries in the currently recognized territories of Venezuela and Colombia (Huertas‐Ramírez & Huertas‐Herrera, 2015; Primo, 1992); this suggests that human‐condor conflict may have a deep history in the region. If genetic erosion is as old as we estimated, this could explain why Andean condors in Venezuela and Colombia were functionally extirpated at the beginning of the 20th century (Calchi & Viloria, 1991; Lieberman et al., 1993), resulting in the fixation of the most common haplotype (I) by the time samples were collected. On the other hand, the estimated bottleneck between the mid‐19th century and 20th century in southern South America matches the time that Andean condors were disappearing from eastern Patagonia (Conway, 2005). Historical observations during the early 19th century suggest that Andean condors were common along the Atlantic coasts. Indeed, during the voyage of the Beagle (1834), Darwin observed breeding pairs of condors in our sampling area (Darwin, 1841) and later scientific expeditions returned to the region describing similar events until the early 20th century (Adams, 1907; Hatcher, 1903). Our finding of a unique mitochondrial lineage in that region suggests that those condors may have represented an endemic coastal subpopulation as first suggested by Lydekker (1895), and not an extension of the distributional range from the mountains due to the abundance of food offered by sheep farms as hypothesized by Adams (1907). Nevertheless, it is possible that the coastal subpopulation was already in decline due to the loss of marine subsidies, forcing condors to feed more on terrestrial mammals (Lambertucci et al., 2018), resulting in a deadly conflict with Patagonian ranchers (Ballejo et al., 2020; Pauli et al., 2018). Paintings and narratives from this period illustrate the persecution of condors, suggesting that hunting was common (e.g., Castellanos, 1923; Darwin, 1841; Lydekker, 1895) and a likely cause behind the contraction of the species distributional range to remote areas in the mountains.

Our bottleneck estimate for the California Condor is consistent with the observational records of condors disappearing from the Pacific coast in the early 20th century (D'Elia et al., 2016). The population crash was so severe that by mid‐20th century only ~150 condors survived in the mountains of California (D'Elia et al., 2016; Snyder & Snyder, 2000). Given that the introduction of cattle in California started back in the 18th century (Chamberlain et al., 2005), it is possible that, as in the case of the Atlantic Andean condors, the collapse of large marine mammal populations during industrial whaling in the 20th century (Rocha et al., 2014) triggered condor's extinction by pushing them into a direct conflict with farmers (Chamberlain et al., 2005; Lotze & Worm, 2009). Although the current recovery of marine mammal populations (Lotze et al., 2011) is a major step forward to re‐establish condor populations in the coasts, for populations to be self‐sustaining, conservation strategies need not only to enhance ecosystem protection (Kurle et al., 2016; Perrig et al., 2020), but also to include appropriate genetic management when considering breeding and translocation programs.

## 5 CONCLUSIONS

Low levels of genetic diversity found in the Andean condor represent a natural state of mtDNA, and thus are unlikely to be an immediate threat to long‐term viability. However, we showed that the detection of further genetic loss is difficult when ancient diversity is already low, highlighting the importance of using museum samples that include the historical range of distribution. We found that similar to California condors, genetic decline of Andean condors correlated with the extinction of the peripheral subpopulations during the introduction of European livestock and collapse of marine subsidies in the last centuries. Given the invariant (and uninformative) nature of mtDNA of Andean condors, we suggest that when possible, rather than translocating individuals from disparate regions, current conservation measures should focus on enhancing gene flow to assist natural augmentation and recolonization. Ideally, future management measures should rely on high‐resolution nuclear genomic data capable of identifying possible cryptic evolutionary units of the species in order to inform which lineages should be used to restock specific geographic locations.

## CONFLICT OF INTEREST

The authors declare that they have no known competing financial interests or personal relationships that could have appeared to influence the work reported in this paper.

## AUTHOR CONTRIBUTION

**Julian Padro:** Conceptualization (equal); Data curation (lead); Formal analysis (lead); Funding acquisition (equal); Investigation (lead); Methodology (lead); Project administration (equal); Resources (equal); Visualization (lead); Writing‐original draft (lead). **Sergio Lambertucci:** Conceptualization (supporting); Funding acquisition (equal); Investigation (equal); Project administration (equal); Resources (lead); Supervision (equal); Writing‐review & editing (equal). **Paula Perrig:** Investigation (supporting); Methodology (supporting); Writing‐review & editing (supporting). **Jonathan Pauli:** Conceptualization (equal); Funding acquisition (equal); Project administration (equal); Resources (lead); Supervision (lead); Writing‐review & editing (lead).

## Supporting information

Supinfo

## Data Availability

Genetic data have been deposited in GenBank under accession number MT993980.
