# Supplementary material for: Andean and California condors possess dissimilar genetic composition but exhibit similar demographic histories
Source: Ecol Evol. 2020 Oct 21;10(23):13011–21. doi: 10.1002/ece3.6887 (PMC7713948; doi:10.1002/ece3.6887)
Supplement: Supplementary file 1 — Supinfo [file ECE3-10-13011-s001.docx]

Supporting Information

Title Andean and California condors possess dissimilar genetic composition but exhibit similar demographic histories

Journal *Ecology & Evolution*

Authors Julian Padró^1,2*^,Sergio A. Lambertucci^1^, Paula L. Perrig^1,2^, Jonathan N. Pauli^2^

Affiliations *^1^ Grupo de Investigaciones en Biología de la Conservación, INIBIOMA, Universidad Nacional del Comahue - CONICET, Quintral 1250 (R8400FRF), Bariloche, Argentina*

*^2^Department of Forest and Wildlife Ecology, University of Wisconsin-Madison, Linden 1630, Madison, WI 53706, USA*

Corresponding Author  *Julian Padró, [padrojulian@gmail.com](mailto:padrojulian@comahue-conicet.gob.ar)*

**Sample Information**

Table S1. Information of DNA samples from 73 Andean condors (mt-Haplotypes: Control region and 12S) grouped by historic and contemporary period (≥< 1961). Details of the DNA sequences can be found in GenBank (accession numbers: AY129644.1-AY129649.1; MT993980).

| Haplotype | Collection date | Period | Country | Locality | Source |
| --- | --- | --- | --- | --- | --- |
| I | 1896 | Historic | Argentina | Santa Cruz | USNM 159965 |
| I | 1897 | Historic | Argentina | Neuquen | MLP 1986 |
| I | 1897 | Historic | Argentina | Santa Cruz | MLP 1987 |
| I | 1898 | Historic | Argentina | Neuquen | MLP 1992 |
| I | 1898 | Historic | Argentina | Neuquen | MLP 1990 |
| I | 1898 | Historic | Argentina | Santa Cruz | FMNH 300615 |
| I | 1898 | Historic | Argentina | Santa Cruz | FMNH 300616 |
| I | 1904 | Historic | Venezuela | Merida | AMNH 469935 |
| I | 1905 | Historic | Chile | Santiago | AMNH 469937 |
| I | 1906 | Historic | Venezuela | Merida | AMNH 469936 |
| I | 1911 | Historic | Argentina | Rio Negro | MVZ 42839 |
| I | 1912 | Historic | Venezuela | Merida | MCZ 92696 |
| I | 1913 | Historic | Ecuador | Mt. Pichincha | AMNH 123924 |
| I | 1913 | Historic | Peru | San Gallen | AMNH 165509 |
| I | 1927 | Historic | Venezuela | Merida | AMNH 469934 |
| I | 1932 | Historic | Peru | Viguata | Hendrickson et al., 2003 |
| I | 1936 | Historic | Colombia | Cauca | FMNH 101049 |
| I | 1938 | Historic | Peru | Ica | LACM 50061 |
| I | 1938 | Historic | Peru | Ica | LACM 50063 |
| I | 1939 | Historic | Argentina | Santa Cruz | AMNH 343883 |
| I | 1939 | Historic | Colombia | Sierra Nevada | AMNH 305595 |
| I | 1946 | Historic | Colombia | Sierra Nevada | USNM 386701 |
| I | 1946 | Historic | Colombia | Sierra Nevada | USNM 386702 |
| I | 1946 | Historic | Colombia | Sierra Nevada | USNM 386703 |
| I | 1946 | Historic | Colombia | Sierra Nevada | USNM 386704 |
| I | 1961 | Contemporary | Argentina | Rio Negro | LACM 56758 |
| I | 1970 | Contemporary | Peru | Ica | UWZM 20284 |
| I | 1970 | Contemporary | Peru | Coastal | Hendrickson et al., 2003 |
| I | 1970 | Contemporary | Peru | Coastal | Hendrickson et al., 2003 |
| I | 1977 | Contemporary | Ecuador | Antisana | Hendrickson et al., 2003 |
| I | 1979 | Contemporary | Argentina | Mendoza | Hendrickson et al., 2003 |
| I | 1996 | Contemporary | Argentina | Mendoza | Hendrickson et al., 2003 |
| I | 1996 | Contemporary | Argentina | Mendoza | Hendrickson et al., 2003 |
| I | 1996 | Contemporary | Argentina | Mendoza | Hendrickson et al., 2003 |
| I | 1996 | Contemporary | Argentina | Mendoza | Hendrickson et al., 2003 |
| I | 1996 | Contemporary | Argentina | Mendoza | Hendrickson et al., 2003 |
| I | 1999 | Contemporary | Ecuador | Antisana | Hendrickson et al., 2003 |
| I | 2008 | Contemporary | Argentina | Rio Negro | CONICET-UW |
| II | 1938 | Historic | Bolivia | Andes | Hendrickson et al., 2003 |
| II | 1979 | Contemporary | Argentina | Mendoza | Hendrickson et al., 2003 |
| II | 1979 | Contemporary | Argentina | Tierra del Fuego | AMNH812848 |
| II | 1983 | Contemporary | Argentina | Mendoza | Hendrickson et al., 2003 |
| II | 1991 | Contemporary | Argentina | Cordoba | Hendrickson et al., 2003 |
| II | 1992 | Contemporary | Argentina | Mendoza | Hendrickson et al., 2003 |
| II | 1992 | Contemporary | Argentina | Mendoza | Hendrickson et al., 2003 |
| II | 1992 | Contemporary | Argentina | Catamarca | Hendrickson et al., 2003 |
| II | 1992 | Contemporary | Argentina | Mendoza | Hendrickson et al., 2003 |
| II | ~1999 | Contemporary | Argentina | Rio Negro | Hendrickson et al., 2003 |
| II | ~1999 | Contemporary | Argentina | Rio Negro | Hendrickson et al., 2003 |
| II | ~1999 | Contemporary | Chile | Santiago | Hendrickson et al., 2003 |
| II | ~1999 | Contemporary | Chile | Santiago | Hendrickson et al., 2003 |
| II | ~1999 | Contemporary | Chile | Santiago | Hendrickson et al., 2003 |
| II | ~1999 | Contemporary | Ecuador | El quiche | Hendrickson et al., 2003 |
| II | 2013 | Contemporary | Argentina | Cordoba | CONICET-UW |
| III | 1977 | Contemporary | Ecuador | Antisana | Hendrickson et al., 2003 |
| III | ~1999 | Contemporary | Ecuador | Cuinca | Hendrickson et al., 2003 |
| III | ~1999 | Contemporary | Ecuador | Pichincha | Hendrickson et al., 2003 |
| IV | 1991 | Contemporary | Argentina | Catamarca | Hendrickson et al., 2003 |
| IV | 2013 | Contemporary | Argentina | Cordoba | CONICET-UW |
| V | 1884 | Historic | Argentina | Santa Cruz | MCZ 41025 |
| V | 1896 | Historic | Argentina | Santa Cruz | USNM 159966 |
| V | 1896 | Historic | Argentina | Santa Cruz | USNM 19485 |
| V | ~1896-1899 | Historic | Argentina | Santa Cruz | USNM 159969 |
| V | 1927 | Historic | Chile | Rio Blanco | LACM 24759 |
| V | 1934 | Historic | Chile | Cordillera | LACM 24760 |
| V | 1934 | Historic | Chile | Curico | FMNH 226437 |
| V | 1934 | Historic | Chile | O’Higgings | FMNH 226438 |
| V | 1994 | Contemporary | Argentina | Rio negro | Hendrickson et al., 2003 |
| V | ~1999 | Contemporary | Chile | Santiago | Hendrickson et al., 2003 |
| V | ~1999 | Contemporary | Chile | Santiago | Hendrickson et al., 2003 |
| V | 2013 | Contemporary | Argentina | Cordoba | CONICET-UW |
| V | 2013 | Contemporary | Argentina | Mendoza | CONICET-UW |
| VI* | 1896 | Historic | Argentina | Santa Cruz | USNM 159968 |

*Novel haplotype: CR partial haplotype 4 (MT993980); 12S partial haplotype 1 (AY129647.1); ~: Approx.

Table S2. Information of DNA samples (mt-Haplotype: D-loop) from 79 California condors (details in D’Elia et al., 2016) grouped by historic and contemporary period (≥< 1961). Details of the haplotype sequences can be found in GenBank (accession numbers: KX379719.1 - KX379736.1).

| Species | Haplogroup | Historic ind. | Contemporary ind. |
| --- | --- | --- | --- |
| *Gymnogyps californianus* | I | 19 | 7 |
|  | II | 1 | - |
|  | III | 1 | - |
|  | IV | 13 | 3 |
|  | V | 3 | - |
|  | VI | 8 | - |
|  | VII | 7 | 4 |
|  | VIII | 3 | - |
|  | IX | 1 | - |
|  | X | 1 | - |
|  | XI | 1 | - |
|  | XII | 1 | - |
|  | XIII | 1 | - |
|  | XIV | 1 | - |
|  | XV | 1 | - |
|  | XVI | 1 | - |
|  | XVII | 1 | - |
|  | XVIII | 1 | - |

**Approximate Bayesian Computation Analysis**

ABC analyses were performed in DYABC 2.1.0 software (Cornuet et al 2014), using two datasets for Andean condors (*Vultur gryphus*) with 12S rRNA (145 bp) and D-loop (165 bp) mitochondrial regions, including 20 historical and 29 contemporary samples from the central-southern region and 15 historical and 9 contemporary samples from northern South America. Dataset of California condors (*Gymnogyps californianus*) comprised 569 bp of D-loop region from 65 historical and 14 contemporary samples from western Mexico and the United States. The mutation model was determined in JModelTest 2.1.10 (Darriba et al 2012), resulting in HKY (Hasegawa, Kishino and Yano 1985) for both Andean (12S and D-loop) and California condors (D-loop; details in Table S3). Prior parameters used for the simulation of demographic scenarios were based on fossil records, historic reports and modern population census of both species (see main text; parameters in Table S4). Computational analysis were performed by comparing within summary statistics (number of haplotypes, segregating sites, mean pairwise differences, private segregating sites and mean of numbers of the rarest nucleotide at segregating sites) and statistics between time periods (number of haplotypes, *F_ST_*, mean pairwise differences within and between samples).

Pre-evaluation of scenario prior combinations showed that our models (parameter prior definitions) are well within the observed data for California and Andean condors (Figure S1). We choose the most likely scenario by comparing the confidence intervals of the probabilities from the posterior distributions using the results of logistic regressions (Cornuet et al 2008; see Table S5). Posterior distribution of parameters was estimated for the most likely scenario using 1% of the total number of simulated data (results in Table S6). Type I error rates were estimated by calculating the average percentage of times the most likely scenario was rejected in favor of alternative scenarios, while Type II errors were assessed by counting decisions in favor of the most likely scenario when it is not the true scenario (Table S7). We used the logistic regression approach testing 1000 pseudo-observed datasets from 1% selected data sets closest to the pseudo-observed data set from the total simulated data (Cornuet et al., 2008; 2014). In addition, we assessed the ‘goodness-of-fit’ by simulating 1% of the datasets from the posterior distribution of parameters (using all summary statistics available in DIYABC 2.1.0), confirming that model specifications used to estimate posterior distributions fitted well within the observed datasets (Figure S2).

Table S3. Mutation parameters used for California condors (*Gymnogyps californianus)* and Andean condors (*Vultur gryphus)*.

| Genetic Parameters | *G. Californianus* (D-loop) | *V. Gryphus* (D-loop) | *V. Gryphus* (12S) |
| --- | --- | --- | --- |
| Mutation model | HKY | HKY | HKY |
| Mean-μ | Uniform (1E-9 - 1E-6) | Uniform (1E-9 - 1E-6) | Uniform (1E-9 - 1E-6) |
| Individual locus-μ | Gamma (1E-9 - 1E-6) | Gamma (1E-9 - 1E-6) | Gamma (1E-9 - 1E-6) |
| Mean K_C/T | Uniform (0.05-20) | Uniform (0.05-20) | Uniform (0.05-20) |
| Individual K_C/T | Gamma (0.05-20) | Gamma (0.05-20) | Gamma (0.05-20) |
| Invariant sites | 0.29 | 0.49 | - |
| Gamma shape | 0.06 | 0.05 | 0.1 |

Table S4. Prior parameters used in ABC analysis to simulate demographic scenarios for California condors and Andean condors.

| Prior | *G. californianus* | *V. gryphus - North* | *V. gryphus - South* | Condition |
| --- | --- | --- | --- | --- |
| Nef1 | U (10 - 500) | U (50 - 1000) | U (500 - 5000) |  |
| Nef2 | U (10 - 50000) | U (50 - 50000) | U (500 - 50000) | >Nef1 |
| Nef3 | U (10 - 500) | U (50 - 1000) | U (100 - 5000) | <Nef1 |
| Nef4 | U (10 - 500) | U (50 - 50000) | U (100 - 50000) | <Nef2 |
| t_1_ | U (1 - 50) | U (1 - 50) | U (1 - 50) |  |
| t_e_ | U (1 - 7142) | U (1 - 7142) | U (1 - 7142) | >t_1_ |
| t_2_ | U (142 - 71428) | U (142 - 71428) | U (142 - 71428) | >t_e_ |

Nef: effective female population size (number of individuals); t: timing of event (in generations); U: Uniformly distributed

Table S5. Comparison of posterior probabilities among scenarios with their 95% confidence intervals, for California condors and Andean condors.

| Scenario/dataset | *G. californianus* | *V. gryphus - North* | *V. gryphus - South* |
| --- | --- | --- | --- |
| Scenario 1 | 0.000 [0.000 - 0.000] | 0.121 [0.116 - 0.126] | 0.002 [0.000 - 0.009] |
| Scenario 2 | 0.432 [0.427 - 0.438] | 0.436 [0.432 - 0.440] | 0.455 [0.447 - 0.463] |
| Scenario 3 | 0.000 [0.000 - 0.000] | 0.161 [0.153 - 0.169] | 0.003 [0.000 - 0.009] |
| Scenario 4 | 0.568 [0.562 - 0.573] | 0.281 [0.279 - 0.284] | 0.540 [0.532 - 0.549] |

Table S6. Demographic parameters estimated from the posterior distribution of the most supported scenario for California and Andean condors.

| Scenario / Dataset | Parameter | Average | Median | Mode | 95% HDPI |
| --- | --- | --- | --- | --- | --- |
| Scenario 4 | Nef1 | 142 | 103 | 37 | 19 - 443 |
| *G. californianus* | Nef2 | 32200 | 33100 | 47400 | 11000 - 49200 |
|  | Nef4 | 14300 | 11900 | 853 | 554 - 39500 |
|  | T1 | 9.05 | 7.28 | 5.89 | 1.27 - 29 |
|  | Te | 3390 | 3300 | 368 | 168 - 69300 |
|  | T2 | 32500 | 30200 | 7500 | 3510 - 69100 |
| Scenario 2 | Nef1 | 503 | 488 | 170 | 75 - 974 |
| *V. gryphus* | Nef2 | 15900 | 12500 | 6760 | 2090 - 45500 |
| North | T1 | 31.2 | 33.2 | 48.2 | 3.9 - 49.6 |
| Scenario 4 | Nef1 | 2320 | 2070 | 855 | 560 - 4710 |
| *V. gryphus* | Nef2 | 39700 | 41700 | 47100 | 18800 - 49500 |
| Central - South | Nef4 | 22700 | 22300 | 33200 | 1370 - 44000 |
|  | T1 | 24.1 | 23.6 | 7.15 | 2.35 - 48.8 |
|  | Te | 2860 | 2620 | 256 | 137 - 6770 |
|  | T2 | 47000 | 50400 | 64700 | 11200 - 70700 |

Nef1: historic-recent effective female population size; Nef2: ancient population size during demographic expansion; Nef3/4: ancient population size during demographic bottleneck; t1: timing of historic-recent bottleneck; te: duration of demographic expansion; t2: timing of ancient bottleneck. Time is in number of generational years (7).

Table S7. Percentage of times the scenario has the highest posterior probability when simulating according to true scenario.

| Successful scenario | True scenario | | | |
| --- | --- | --- | --- | --- |
| *G. californianus* | 1 | 2 | 3 | 4 |
| 1 | 73.1 | 66.2 | 8.3 | 5.8 |
| 2 | 2.4 | 1.5 | 30.8 | 23.6 |
| 3 | 24.5 | 32.3 | 10 | 5.6 |
| 4 | 0 | 0 | 50.9 | 65.0 |
| *V. gryphus - North* | 1 | 2 | 3 | 4 |
| 1 | 75.6 | 8.3 | 67.5 | 3.1 |
| 2 | 3.8 | 39.1 | 5.1 | 26.2 |
| 3 | 20.6 | 7.3 | 27.4 | 4.2 |
| 4 | 0 | 45.3 | 0 | 66.5 |
| *V. gryphus - South* | 1 | 2 | 3 | 4 |
| 1 | 0 | 0 | 0 | 0 |
| 2 | 9.1 | 18.0 | 8.4 | 17.3 |
| 3 | 83.0 | 30.6 | 80.3 | 21.5 |
| 4 | 7.9 | 51.4 | 11.3 | 61.2 |


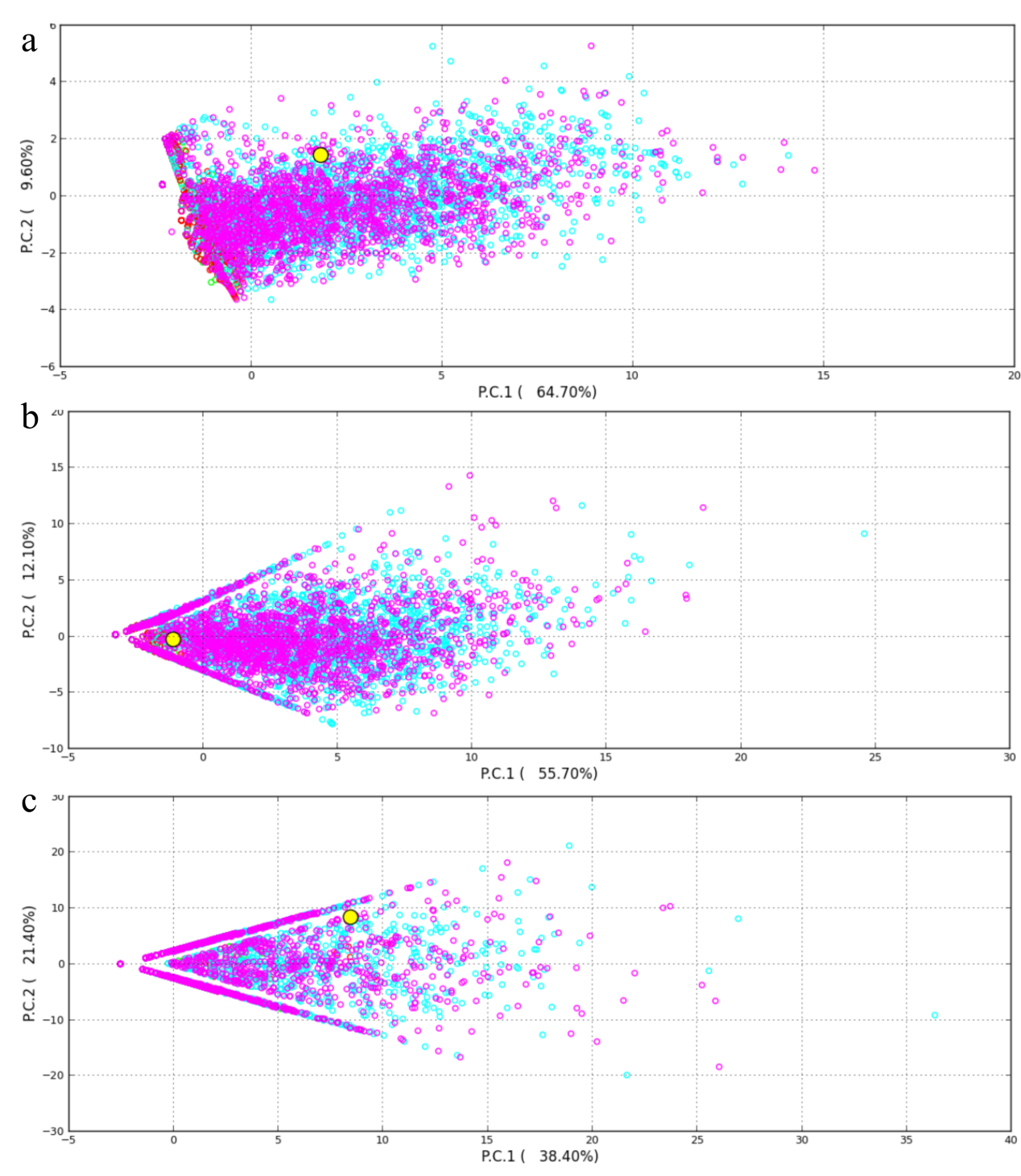


Figure S1. Principal Component Analysis of scenario-prior combinations for California (a) and Andean condors from north (b) and central-south region (c). Small green, purple, red and blue dots represent simulated scenarios 1, 2, 3 and 4, respectively, while the large yellow dot represents the observed data set.


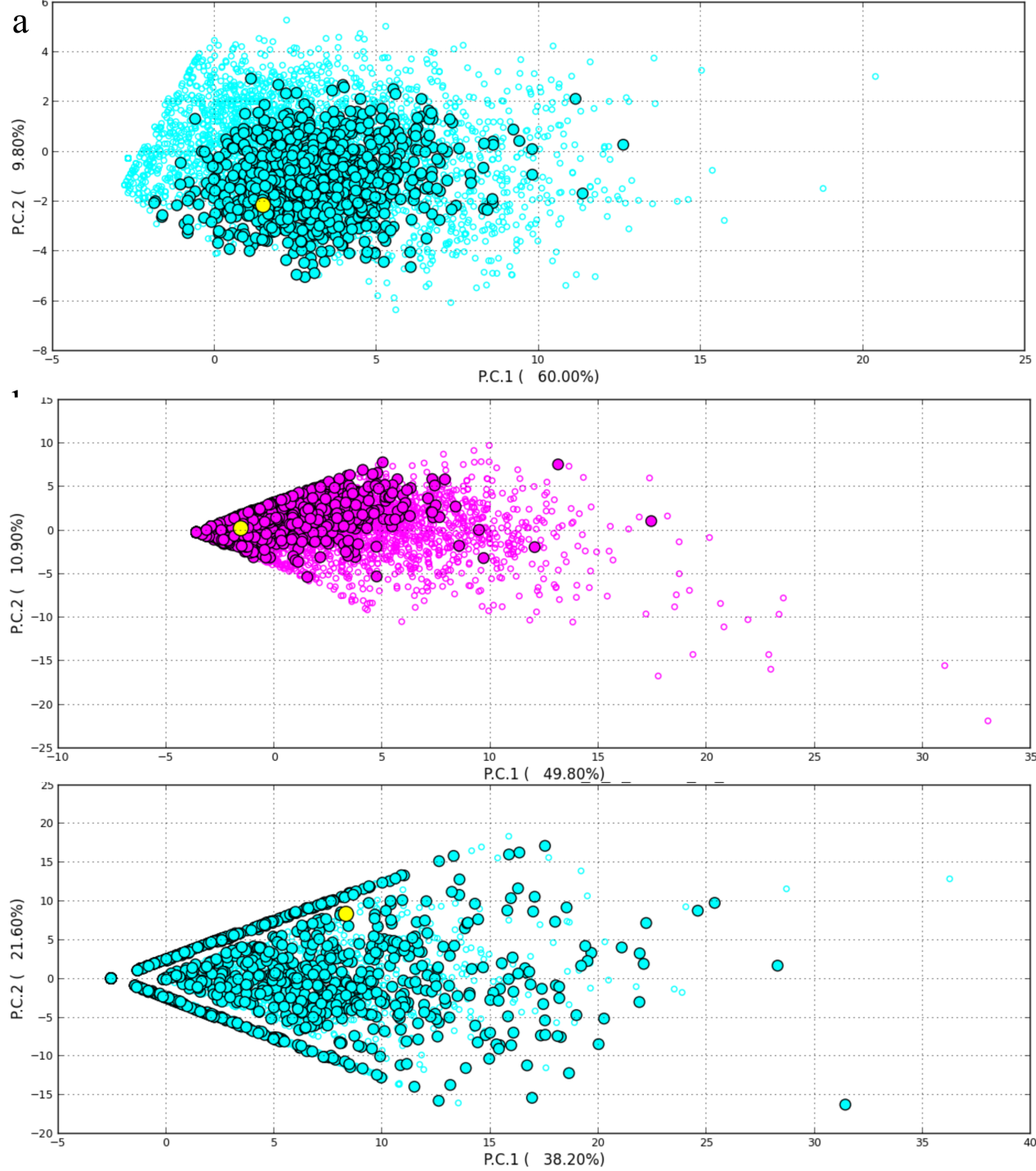


Figure S2. Principal Component Analysis of scenario-prior and posterior combinations of parameters for California (a) and Andean condors from north (b) and central-south region (c). Empty and solid dots represent datasets simulated from the prior and posterior distributions for the most supported scenario (Blue: scenario 4; Purple: scenario 2), whereas yellow dots represent the observed data.

**References**

Cornuet, J. M., Santos, F., Beaumont, M. A., Robert, C. P., Marin, J. M., Balding, D. J., ... & Estoup, A. (2008). Inferring population history with DIY ABC: a user-friendly approach to approximate Bayesian computation. Bioinformatics, 24(23), 2713-2719.

Cornuet, J. M., Pudlo, P., Veyssier, J., Dehne-Garcia, A., Gautier, M., Leblois, R., ... & Estoup, A. (2014). DIYABC v2. 0: a software to make approximate Bayesian computation inferences about population history using single nucleotide polymorphism, DNA sequence and microsatellite data. Bioinformatics, 30(8), 1187-1189.

Darriba, D., Taboada, G. L., Doallo, R., & Posada, D. (2012). jModelTest 2: more models, new heuristics and parallel computing. Nature methods, 9(8), 772.

D'Elia, J., Haig, S. M., Mullins, T. D., & Miller, M. P. (2016). Ancient DNA reveals substantial genetic diversity in the California Condor (Gymnogyps californianus) prior to a population bottleneck. The Condor: Ornithological Applications, 118(4), 703-714.

Hasegawa M, Kishino H, Yano T (1985). "Dating of human-ape splitting by a molecular clock of mitochondrial DNA". Journal of Molecular Evolution. 22 (2): 160–174.

Hendrickson, S. L., Bleiweiss, R., Matheus, J. C., de Matheus, L. S., Jácome, N. L., & Pavez, E. (2003). Low genetic variability in the geographically widespread Andean Condor. The Condor, 105(1), 1-12.
